# Case Report: Management challenges of active tuberculosis complicated by thrombocytosis and squamous cell lung carcinoma

**DOI:** 10.3389/fonc.2026.1723876

**Published:** 2026-03-10

**Authors:** Chen Zhu, Yanli Xu, XiaoYing Li, HongBao Ma, Tingshu Jiang

**Affiliations:** 1 Department of Respiratory and Critical Care Medicine, Yantai Yuhuangding Hospital, Shandong, Yantai, China; 2 Department of Infectious Diseases, Yantai Qishan Hospital, Shandong, Yantai, China; 3 Department of Medical Affairs, Yantai Qishan Hospital, Shandong, Yantai, China; 4 Department of Tuberculosis, Yantai Qishan Hospital, Shandong, Yantai, China

**Keywords:** active tuberculosis, anti-tuberculosis therapy, anti-tumor treatment, squamous cell lung carcinoma, thrombocytosis

## Abstract

**Background:**

This report assesses the feasibility of a sequential treatment strategy for a patient with active tuberculosis (TB) complicated by thrombocytosis and squamous cell lung carcinoma, involving initial anti-TB therapy combined with aspirin, followed by anticancer treatment. It highlights the challenge of balancing infection control, hematologic stability, and oncologic management in this complex scenario.

**Case presentation:**

A 55-year-old man with a 15-day history of cough, sputum, and fever was found to have markedly elevated platelet levels. Bronchoscopy confirmed active TB with concurrent lung cancer. Following anti-TB therapy combined with aspirin for antiplatelet management, platelet counts returned to normal. One month later, antitumor chemotherapy with nab-paclitaxel and carboplatin was initiated, leading to effective disease control.

**Conclusion:**

Active TB complicated by thrombocytosis and squamous cell lung carcinoma poses diagnostic and therapeutic challenges. This case demonstrates that early anti-TB therapy combined with aspirin can effectively normalize platelet counts and facilitate the timely initiation of anticancer treatment.

## Background

Tuberculosis (TB) caused by *Mycobacterium tuberculosis* (MTB) primarily affects the lungs and remains a leading cause of infectious mortality worldwide. According to the WHO Global Tuberculosis Report 2024, there were an estimated 10.6 million people who fell ill with TB worldwide in 2023, and 1.3 million deaths from TB in the same year. In China, 741,000 new TB cases were estimated in 2023, ranking third among the 30 high TB burden countries and accounting for 6.8% of the global incidence (1). Platelet counts in patients with active TB are significantly higher than in those with subclinical TB (2). TB is also a risk factor for lung cancer, with the two diseases closely linked and often aggravating each other, thereby posing a growing public health challenge (3). Lung cancer is histologically categorized into non-small cell lung cancer (NSCLC) and small cell lung cancer (SCLC). NSCLC encompasses several subtypes, including squamous cell carcinoma (SCC)—the variant presented in this case. Tobacco smoking remains the predominant risk factor for pulmonary SCC. Diagnosis relies on imaging techniques and histopathological confirmation, while treatment strategies include surgery, chemotherapy, radiotherapy, targeted therapy, and immunotherapy, depending on disease stage and molecular characteristics. This report describes the clinical presentation and management of a patient with TB complicated by thrombocytosis and squamous cell lung carcinoma, and reviews relevant literature.

## Case presentation

A 55-year-old man presented with a 2-week history of cough with white mucoid sputum and fever up to 38.4°C. He reported a significant smoking history of 20 pack-years (1 pack per day for 20 years) but denied any history of recent infections, surgery, trauma, alcohol abuse, or occupational exposure to dust, asbestos, or other carcinogens.

**Figure 1:**
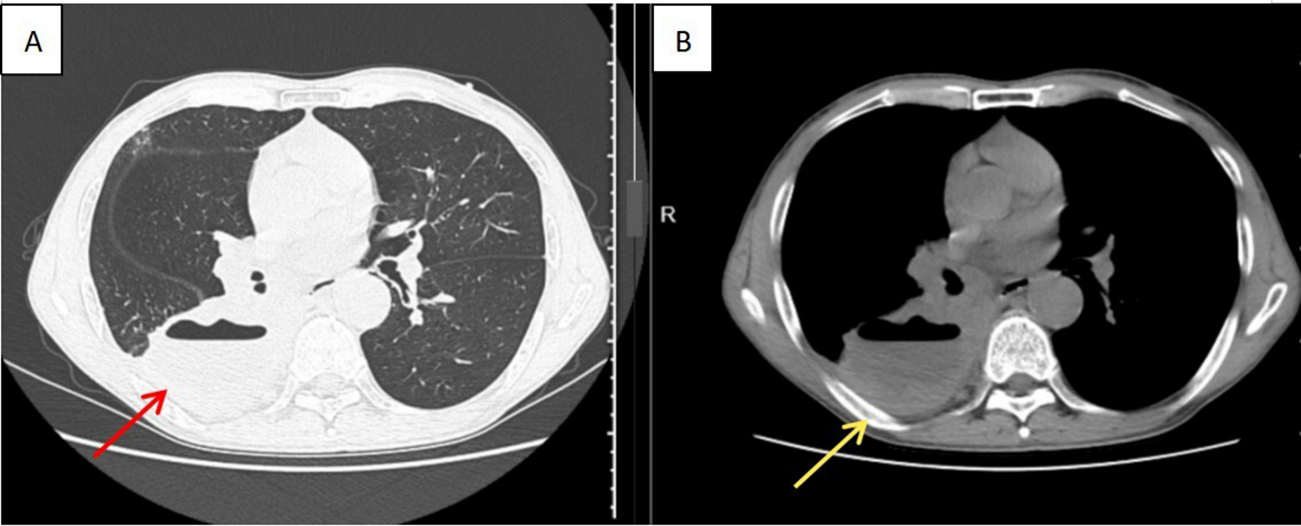
Enhanced chest CT demonstrated a right lung mass with mediastinal and right hilar lymphadenopathy. **(A)** (lung window) clearly delineates the morphology and margins of the lesion within the pulmonary parenchyma; **(B)** (mediastinal window) shows the enlarged mediastinal lymph nodes.

On admission, vital signs were stable: temperature, 36.6°C; pulse, 70/min; respiratory rate, 18/min; and blood pressure, 110/64 mmHg. Lung auscultation showed diminished breath sounds in the right lung and coarse breath sounds in the left. Contrast-enhanced chest CT demonstrated a right lung mass suspicious for malignancy with possible infection or abscess, and mediastinal and right hilar lymphadenopathy suggestive of metastasis (**Figure 1**). He was initially diagnosed with a pulmonary space-occupying lesion.

Laboratory evaluation revealed white blood cells (WBC), 10.38 × 10^9^/L; hemoglobin, 128 g/L; platelets, 1,054 × 10^9^/L; plateletcrit, 0.69; mean platelet volume (MPV), 6.5 fL; platelet distribution width (PDW), 14.8%; large platelet ratio, 5.1; high-sensitivity C-reactive protein (hs-CRP), 43.63 mg/L; procalcitonin, 0.064 ng/L; alanine aminotransferase (ALT), 65.9 U/L; albumin (ALB), 29 g/L; and total protein (TP), 61.7 g/L. Arterial blood gas showed pH 7.42; partial pressure of carbon dioxide (PaCO_2_), 40.98 mmHg; partial pressure of oxygen (PaO_2_), 76.95 mmHg; and lactate, 1.48 mmol/L. Sputum specimen examinations were negative for both acid-fast bacillus (AFB) smear microscopy and MTB culture.

**Figure 2:**
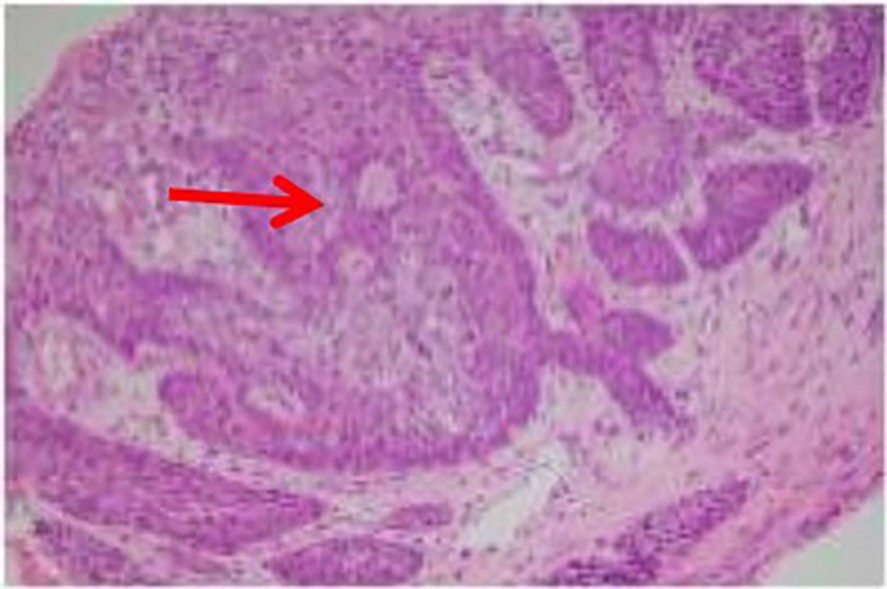
Histopathological examination confirmed moderately differentiated squamous cell carcinoma of the lung. Hematoxylin–eosin (HE) staining (×200 magnification) revealed characteristic nests of polygonal tumor cells with abundant eosinophilic cytoplasm and distinct cell borders.

He was admitted with the diagnoses of right pulmonary space-occupying lesion, mediastinal lymphadenopathy, and thrombocytosis. On day 2 of admission, fiberoptic bronchoscopy revealed external compression of the right middle lobe medial segment, an obstructing endobronchial lesion with necrotic debris in the right lower lobe dorsal segment, and mucosal congestion and edema in the basal segments. Biopsy was performed in the dorsal segment, and bronchoalveolar lavage was obtained from the basal segments. Endobronchial ultrasound (EBUS) revealed enlarged mediastinal lymph nodes at stations 4R, 7, and 10R, which were sampled by needle aspiration (**Supplementary Figure 1**). Nanopore sequencing of bronchoalveolar lavage fluid (BALF) detected MTB (137 sequences), and pathology confirmed SCC (**Figure 2**). Programmed death-ligand 1 (PD-L1) expression in lung tissue tested negative. Positron emission tomography–computed tomography (PET–CT) demonstrated right lung carcinoma with bilateral pulmonary metastases, multiple lymph node metastases, and right pleural metastases with increased uptake (**Supplementary Figure 2**). Magnetic resonance imaging (MRI) of the brain revealed multiple ischemic foci and left maxillary sinusitis.

**Figure 3:**
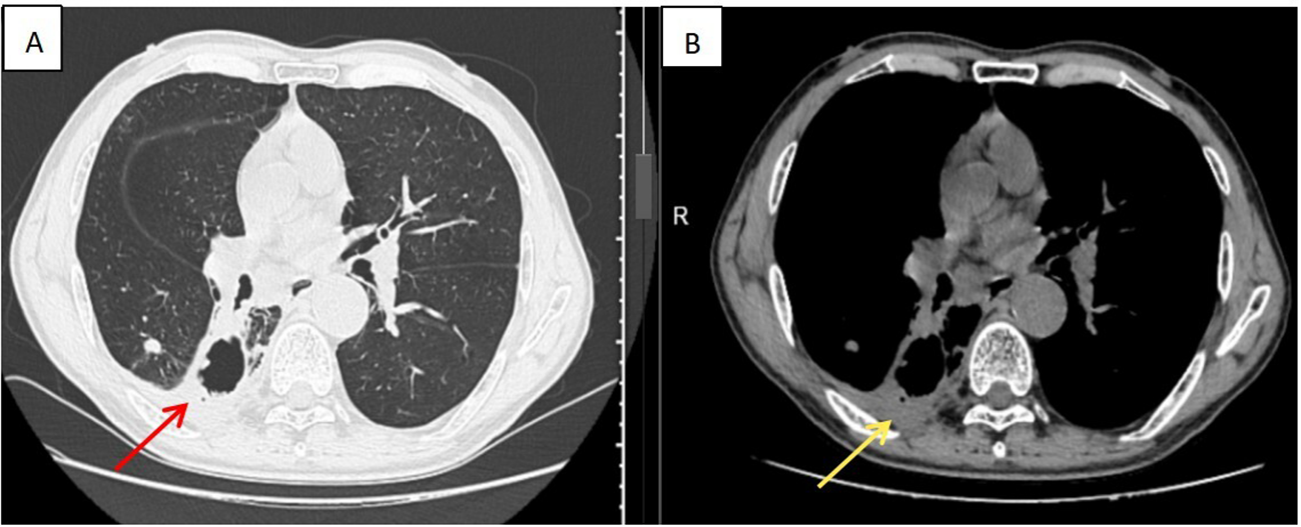
Follow-up chest CT on 6 September 2025 demonstrated significant reduction in the size of the right lung lesion (arrow), measuring 3.2 × 3.1 cm. **(A)** Red arrow: lung window, **(B)** yellow arrow: mediastinal window.

**Figure 4:**
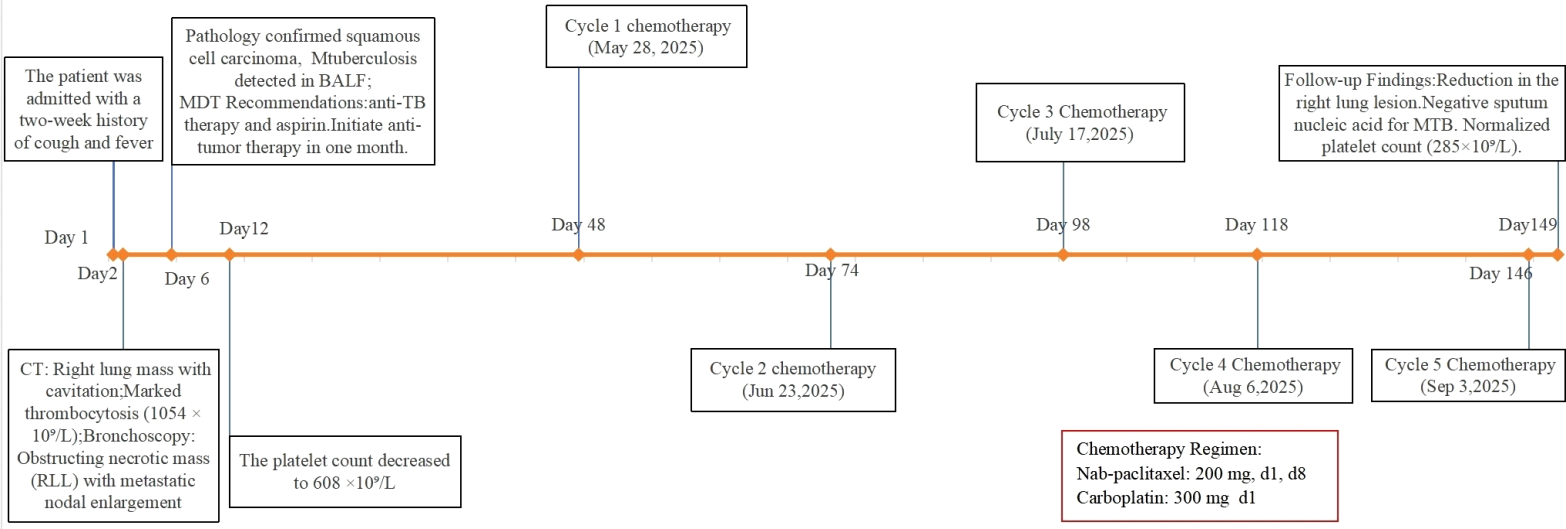
Clinical course timeline.

The patient was diagnosed with moderately differentiated SCC of the right lung (clinically staged as cT4N3M1a, Stage IVA), concurrent with active pulmonary TB and thrombocytosis. No prior history of anti-TB therapy was reported. A multidisciplinary team comprising oncology, thoracic surgery, pulmonology, and interventional medicine recommended initiation of anti-TB therapy combined with aspirin as antiplatelet management. The patient weighed 70 kg, and the anti-TB regimen comprised rifampicin 450 mg once daily, isoniazid 300 mg once daily, pyrazinamide 750 mg twice daily (administered in divided doses to improve tolerability), and ethambutol 750 mg once daily along with aspirin 100 mg once daily. A follow-up complete blood count on day 12 revealed a significant decrease in the platelet count, from 1,054 × 10^9^/L to 608 × 10^9^/L. After 1 month, laboratory evaluation revealed platelets reduced to 328 × 10^9^/L, MPV 8.0 fL, PDW 7.9%, and large platelet ratio 10.3%, but liver enzymes were elevated (ALT 107 U/L, AST 103 U/L, and GGT 66 U/L), attributed to anti-TB medications. Hepatoprotective therapy (bifendate 50 mg tid and Ganshuang granules 3 g tid) was initiated, liver function subsequently improved (ALT 44.9 U/L, AST 32.8 U/L, and GGT 64 U/L), and the original anti-TB regimen was retained. A follow-up chest CT prior to chemotherapy showed significant improvement of the pulmonary infiltrates compared to the previous one. Chemotherapy was initiated on day 48 with nab-paclitaxel 200 mg (d1, d8) plus carboplatin 300 mg (d1). After 2 months of intensive therapy, the anti-TB regimen was modified to rifapentine 450 mg biw, moxifloxacin 400 mg qd, and isoniazid 450 mg qd. This therapeutic modification aligned with the optimized treatment regimen for drug-susceptible TB recommended by the World Health Organization (WHO) (4). The patient subsequently received additional chemotherapy cycles on June 23, July 17, August 6, and September 3 (cycles 2–5). On day 149 after admission, follow-up chest CT showed reduction in the right lung lesion (**Figure 3**). Sputum nucleic acid testing for MTB was negative. Platelet count normalized to 285 × 10^9^/L; MPV, 6.9 fL; PDW, 15.4%; and large platelet ratio, 7.7%. Liver function was normal (ALT, 22.6 U/L and AST, 20.5 U/L). The patient treatment timeline is shown in **Figure 4**.

## Discussion

Studies indicate that in active pulmonary TB, platelet counts often decline after anti-TB therapy, a change associated with sputum conversion and favorable outcomes, making platelet count a useful marker for treatment response (5–7). Significant differences are observed when comparing platelet levels before and after 2 months of therapy, particularly in patients with elevated baseline counts, nearly half of whom show reductions by the end of the intensive phase (5). Platelet count has also been proposed as an independent predictor to differentiate TB from non-tuberculous pneumonia (8). Reta et al. reported hematological changes, including platelets, after therapy initiation (9). Excessive thrombocytosis may raise the risk of ischemic stroke, with most cases occurring within 3 months of TB diagnosis due to immune-driven platelet hyperactivation (10). The patient was prescribed low-dose aspirin (100 mg daily) for antiplatelet therapy. This dosage is standard in secondary cardiovascular prevention, and its safety in older adults is corroborated by recent clinical analyses (11). Furthermore, support for using aspirin in this inflammatory context comes from a published case where it successfully managed reactive thrombocytosis secondary to dengue fever (12). Aspirin has shown multiple benefits in pulmonary TB, including improved survival, reduced mortality, higher sputum conversion, suppressed cytokine secretion, and decreased cavity burden (13, 14). The marked thrombocytosis in this case posed a diagnostic challenge, as it could be attributed to either a paraneoplastic syndrome from the advanced lung cancer or a reactive process secondary to active TB. The rapid decline in platelet count observed as early as day 7 of anti-TB therapy, prior to any anticancer treatment, strongly suggests that the systemic inflammation from TB was the primary driver. However, a contributory role from the malignancy cannot be entirely excluded, as both conditions can stimulate thrombopoiesis through overlapping inflammatory pathways. Low-dose aspirin administered concomitantly with the initial HRZE regimen was pivotal for managing thrombocytosis. Aspirin served a dual role: as an irreversible COX-1 inhibitor, it reduced platelet aggregation and thrombotic risk (10); additionally, its anti-inflammatory effect—partly via NF-κB inhibition—likely tempered the TB-driven systemic inflammation that promotes reactive thrombocytosis. The rapid platelet reduction before chemotherapy highlights the thrombocytosis’ inflammatory origin and demonstrates how anti-TB therapy plus aspirin safely bridged the patient to anticancer treatment by stabilizing hematologic parameters.

The risk of lung cancer is significantly increased in patients with TB, particularly those with smoking or chronic pulmonary diseases, implicating TB-associated chronic inflammation in carcinogenesis (15). Tobacco smoking serves as a well-established primary driver of pulmonary SCC. In this case, a cavitary lesion initially suggestive of TB was confirmed as SCC by bronchoscopic biopsy. This highlights the importance of considering lung cancer in patients with TB with high-risk factors such as advanced age and heavy smoking, even when imaging appears typical of TB. Most studies suggest that combining first-line anti-TB drugs with chemotherapy does not cause major additional toxicity or interactions (16), though immunosuppression during chemotherapy may trigger new or latent TB infections (17), warranting monitoring with MTB nucleic acid tests and imaging. Chronic inflammation, abnormal molecular expression, genomic alterations, and fibrosis from TB infection contribute to lung cancer incidence and mortality (18, 19). However, potential drug interactions warrant attention, particularly with rifampicin—a potent inducer of cytochrome P450 (CYP) enzymes and P-glycoprotein. In this case, the use of nab-paclitaxel (less dependent on CYP metabolism) and carboplatin (renally cleared) likely minimized interactions with anti-TB drugs. This regimen is also supported by its established efficacy in SCC (20). Nevertheless, rifampicin may reduce anticancer drug levels, compromising efficacy. Thus, therapeutic drug monitoring, dose adjustment, or substitution with rifabutin should be considered in such scenarios. In NSCLC with TB, effective anti-TB treatment for 2–4 weeks permits safe initiation of antitumor therapy, including immune checkpoint inhibitors (ICIs) (21), although ICIs may increase TB risk and reactivate latent infection (22). Rifampicin and isoniazid inhibit MTB by inducing iNOS expression to enhance nitric oxide (NO) production in macrophages (23–26). The pathogen counteracts this by suppressing NOS2/NO via the PPE protein and IL-10/STAT3 pathway—a signaling axis that also promotes tumor progression (23–27). This shared mechanism may explain the higher drug resistance observed in patients with concurrent TB and lung cancer and suggests that certain anticancer drugs could be repurposed for TB therapy (28).

In this case, hepatoprotective therapy using bifendate and Ganshuang granules was employed. This approach reflects the synergistic role of traditional Chinese medicine in mitigating anti-TB drug-induced hepatotoxicity and improving liver function, providing a reference for the clinical management of drug-induced liver injury.

In conclusion, active TB with thrombocytosis and lung cancer is a rare but challenging condition with overlapping diagnostics and therapeutic conflicts. For patients with active TB co-existing with lung cancer and thrombocytosis, treatment decisions require comprehensive consideration of infection control and thrombotic risk. Management typically prioritizes controlling tuberculous infection, with antitumor therapy initiated sequentially after clinical stabilization. When concurrent treatment is necessary, it should be cautiously implemented under adequate anti-TB protection. In this process, low-dose aspirin serves as a safe and effective approach for managing thrombocytosis, while multidisciplinary team collaboration plays a pivotal role in balancing anti-infection, antitumor, and thromboprophylaxis strategies. However, as this is a single case, the findings are limited and larger studies are required to validate optimal strategies.

## Data Availability

The original contributions presented in the study are included in the article/**Supplementary Material**. Further inquiries can be directed to the corresponding authors.

## Supplementary material

Supplementary Figure 1 **(A)** Fiberoptic bronchoscopy shows an intraluminal mass with whitish necrotic material in the dorsal segment of the right lower lobe. **(B)** Endobronchial ultrasound (EBUS) reveals enlarged mediastinal lymph nodes.

Supplementary Figure 2 PET-CT imaging findings: **(A)** Mediastinal window demonstrates the primary right lung carcinoma (8.7 × 5.9 cm). **(B)** Sagittal and **(C)** coronal views confirm multiple lymph node metastases, all showing significantly increased metabolic uptake.
