# Supplementary figures and images for: Case Report: Management challenges of active tuberculosis complicated by thrombocytosis and squamous cell lung carcinoma
Source: Front Oncol. 2026 Mar 10;16:1723876. doi: 10.3389/fonc.2026.1723876 (PMC13008696; doi:10.3389/fonc.2026.1723876)

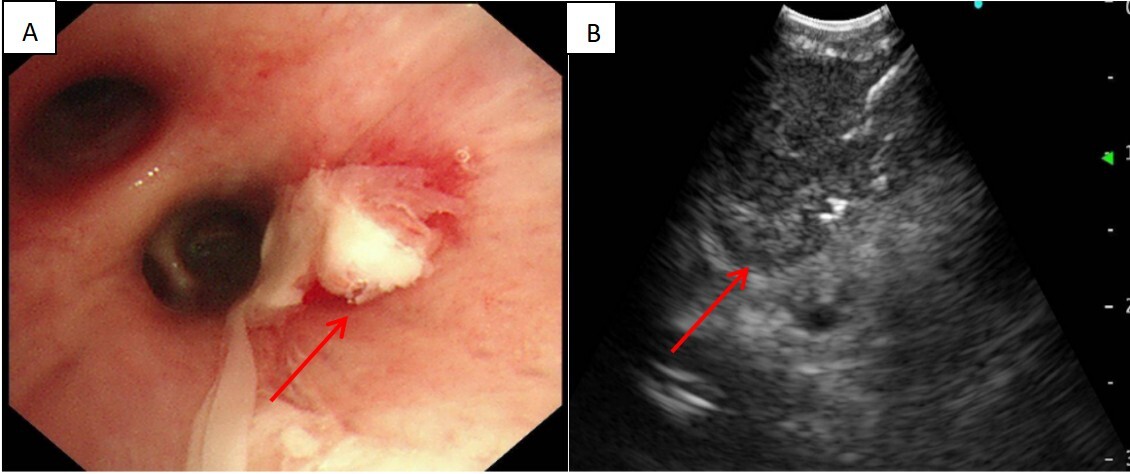

Supplement: Supplementary Figure 1 — (A) Fiberoptic bronchoscopy shows an intraluminal mass with whitish necrotic material in the dorsal segment of the right lower lobe. (B) Endobronchial ultrasound (EBUS) reveals enlarged mediastinal lymph nodes. [file Image1.jpeg]

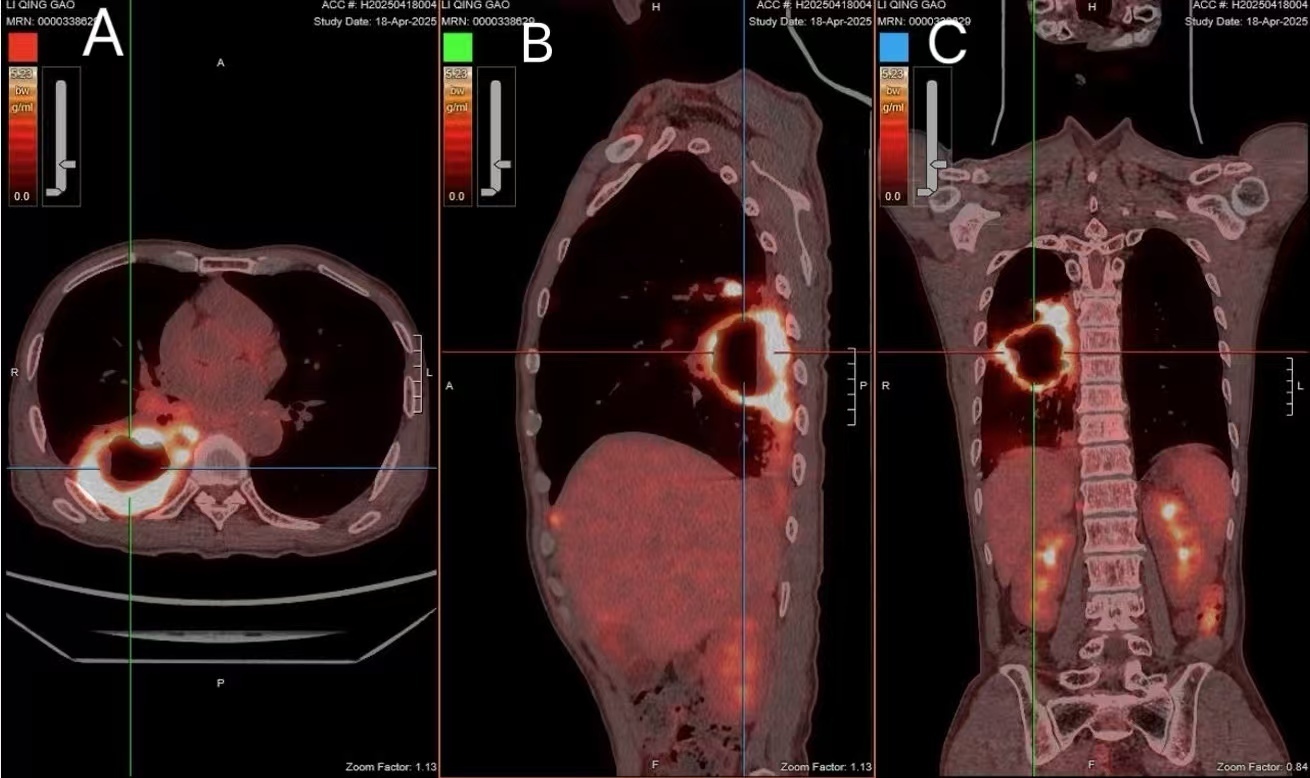

Supplement: Supplementary Figure 2 — PET-CT imaging findings: (A) Mediastinal window demonstrates the primary right lung carcinoma (8.7 × 5.9 cm). (B) Sagittal and (C) coronal views confirm multiple lymph node metastases, all showing significantly increased metabolic uptake. [file Image2.jpeg]
